# The Combined Intervention with Germinated *Vigna radiata* and Aerobic Interval Training Protocol Is an Effective Strategy for the Treatment of Non-Alcoholic Fatty Liver Disease (NAFLD) and Other Alterations Related to the Metabolic Syndrome in Zucker Rats

**DOI:** 10.3390/nu9070774

**Published:** 2017-07-19

**Authors:** Garyfallia Kapravelou, Rosario Martínez, Elena Nebot, María López-Jurado, Pilar Aranda, Francisco Arrebola, Samuel Cantarero, Milagros Galisteo, Jesus M. Porres

**Affiliations:** 1 Department of Physiology, Institute of Nutrition and Food Technology, Centre for Biomedical Research, Institute of Health and Sport, University of Granada, 18071 Granada, Spain; kapravelou@yahoo.gr (G.K.); rosario.mzmz@gmail.com (R.M.); enebot@ugr.es (E.N.); mlopezj@ugr.es (M.L.-J.); paranda@ugr.es (P.A.); 2 Department of Histology, Institute of Neurosciences, Centre for Biomedical Research, University of Granada, 18071 Granada, Spain; fav@ugr.es; 3 Centro de Instrumentación Científica, University of Granada, Campus Universitario de Fuentenueva s/n, 18071 Granada, Spain; ascm@ugr.es; 4 Department of Pharmacology, School of Pharmacy, University of Granada, Campus Universitario de Cartuja s/n, 18071 Granada, Spain; mgalist@ugr.es

**Keywords:** non-alcoholic fatty liver disease, metabolic syndrome, *Vigna radiata*, legume protein hydrolyzate, aerobic interval training, diet, germination, obesity, exercise, liver histology

## Abstract

Metabolic syndrome (MetS) is a group of related metabolic alterations that increase the risk of developing non-alcoholic fatty liver disease (NAFLD). Several lifestyle interventions based on dietary treatment with functional ingredients and physical activity are being studied as alternative or reinforcement treatments to the pharmacological ones actually in use. In the present experiment, the combined treatment with mung bean (*Vigna radiata*), a widely used legume with promising nutritional and health benefits that was included in the experimental diet as raw or 4 day-germinated seed flour, and aerobic interval training protocol (65–85% VO_2_ max) has been tested in lean and obese Zucker rats following a 2 × 2 × 2 (2 phenotypes, 2 dietary interventions, 2 lifestyles) factorial ANOVA (Analysis of Variance) statistical analysis. Germination of *V. radiata* over a period of four days originated a significant protein hydrolysis leading to the appearance of low molecular weight peptides. The combination of 4 day-germinated *V. radiata* and aerobic interval training was more efficient compared to raw *V. radiata* at improving the aerobic capacity and physical performance, hepatic histology and functionality, and plasma lipid parameters as well as reverting the insulin resistance characteristic of the obese Zucker rat model. In conclusion, the joint intervention with legume sprouts and aerobic interval training protocol is an efficient treatment to improve the alterations of glucose and lipid metabolism as well as hepatic histology and functionality related to the development of NAFLD and the MetS.

## 1. Introduction

Metabolic syndrome (MetS) is a group of metabolic alterations characterized by central obesity, dyslipidemia, elevated plasma glucose, insulin resistance, elevated blood pressure, pro-thrombotic and pro-inflammatory state [1]. Patients with MetS are also more susceptible to develop type 2 diabetes mellitus and suffer increased risk of developing cardiovascular disease. In addition, their hepatic morphology and functionality can be adversely affected leading to the development of Non Alcoholic Fatty Liver Disease (NAFLD). Such disease has been historically regarded as the liver manifestation of MetS although recent studies have concluded that is one of the major risk factors that strongly determines its progression [2,3]. Hepatic morphology and functionality in NAFLD is adversely affected and can be categorized in four different stages starting from a simple steatosis (liver fat deposition and mild inflammation), the development of Non alcoholic steatohepatitis (NASH) that includes steatosis plus inflammation and hepatocyte “ballooning”, the appearance of fibrosis and, in some cases, can lead to the last stage of hepatocellular carcinoma (HCC) [4]. Although the exact mechanisms leading to NAFLD are not yet completely understood, insulin resistance, hormones secreted from the adipose tissue, nutritional factors, gut microbiota and genetic and epigenetic factors have been described to play a major role [5].

The development of strategies for the prevention and treatment of MetS includes changes in lifestyle such as caloric restriction, low fat and low glycemic index diets, consumption of foods rich in beneficial bioactive ingredients, and regular physical activity [1,6]. In this regard, legumes exhibit a variety of health effects related to their antioxidant, hypoglycemic, and hypolipidemic properties [7,8] due to the specific characteristics of legume proteins, carbohydrates and lipids, and also from several non-nutritional compounds like polyphenols, phytic acid or α-galactoside oligosaccharides. Germination and fermentation are alternative biotechnological treatments that render protein preparations with important degree of hydrolysis and bioactive compounds with beneficial health actions [9,10]. The resulting bioactive peptides may act as potential physiological modulators of metabolism, given that they inhibit the activity of angiotensin converting enzyme and show antioxidant and bile acid-binding properties [11], thus showing promising potential as functional ingredients. *Vigna radiata* is a widely used legume for human consumption that exhibits an interesting composition of nutrients and bioactive compounds [12,13]. Such nutritive value can be significantly improved by technological processing like sprouting [14]. In fact, *V. radiata* sprouts are usually commercialized to take part in numerous healthy dishes that are becoming increasingly popular for the general consumer.

The effects of different types of exercise on the treatment of MetS have been studied concerning the cardiovascular risk, and high-intensity aerobic interval training has been reported to be more effective at reducing such risk in rats with MetS than moderate-intensity continuous training did [15]. With regard to liver metabolism, several authors have studied the beneficial effects of moderate or vigorous intensity exercise on different aspects of NAFLD [6,16].

Different in vivo experimental models have been used to test the beneficial effects of diet and exercise on several parameters of MetS. Among them, the obese Zucker rat is a widely accepted animal experimental model to study the multifactorial effects of diet and exercise on MetS associated conditions. This rat strain is known to present a genetic defect in leptin receptor that causes the development of hyperphagia leading to obese phenotype [17], and shares many similarities with humans affected by MetS. [18]. We have previously demonstrated the beneficial effects of an aerobic interval training (AIT) protocol on NAFLD, insulin resistance and other features related to the development of MetS in the obese Zucker rat model [6].

The aim of this study was to assess the effects on glucose and lipid metabolism parameters, as well as on liver histology and functionality, exhibited by a novel joint intervention combining the consumption of 4 day-germinated *V. radiata* as dietary source of bioactive compounds and hydrolyzed protein obtained using natural methods, together with an AIT protocol. Such joint intervention (diet plus exercise) in the above mentioned experimental animal model of the obese Zucker rat was an efficient strategy to improve the NAFLD condition related to the development of MetS.

## 2. Materials and Methods

### 2.1. Vigna radiata Seeds and Germination Process

*Vigna radiata* L. (La Asturiana, León) seeds were obtained from a commercial establishment and powdered (0.18 mm sieve) for sample analysis. Sprouting was done in seeds previously sterilized by immersion in sodium hypochlorite for 3 min and washed out with sterilized, type-2 water to eliminate any traces of sodium hypochlorite. The seeds were then left soaking in water during 8 h and distributed in trays over sheets of filter paper where they were left covered in darkness, at 30 °C, during 4 days. After this period, sprouted *V. radiata* seeds were collected, milled and lyophilized. Once lyophilized, the sprouts were milled again to a fine powder (0.18 mm sieve) for sample analysis and diet preparation.

### 2.2. Composition Analysis of Raw and Germinated V. radiata Flours and Experimental Diets

Moisture content of the raw (RVR) and 4 day-germinated (GVR) *V. radiata* flours and experimental diets was determined by drying to constant weight in an oven at 105 ± 1 °C. Total N was determined according to Kjeldahl’s method. Crude protein was calculated as N × 6.25. Insoluble N and soluble protein and non-protein N of the legume flours were measured as described by Kapravelou et al. [7]. Samples (0.5 g) were thrice extracted with 10 mL of 0.02N NaOH during 45 min and the three extracts pooled. Insoluble material was removed by centrifugation at 3000 rpm for 20 min. The supernatant was mixed with 20 mL of 30% trichloroacetic acid and the mixture stirred for 15 min at 4 °C. Protein was removed by centrifugation at 3000 rpm for 15 min. Total N was measured in the insoluble material (insoluble N), protein pellet (protein N) and supernatant (non protein N). Total fat content was determined by gravimetry of the ether extract after acid hydrolysis of the sample. Ash content was measured by calcination at 450 °C to a constant weight.

### 2.3. Sodium Dodecyl Sulfate-Polyacrylamide Gel Electrophoresis (SDS-PAGE)

Was done according to the method of Laemmli [19]. The final concentration of acrylamide in the running gel was 14%. Equal amounts of N (2.0 µg) were loaded in each lane. The gels were fixed and stained with Coomassie brilliant blue R-250 (EZBlue, Sigma, St. Louis, MO, USA). The mixture of molecular weight markers ranged 8–220 KDa (ColorBurst^TM^, Sigma, St. Louis, MO, USA).

### 2.4. Mass Spectrophotometry

An Ultra Performance Liquid Chromatography (UPLC) (Acquity H Class, Waters Corporation, Manchester, UK) coupled by quadrupole-time-of-flight mass spectrometry (Synap G2, Waters Corporation, Manchester, UK) was employed for all high-resolution mass spectrometry analysis. Prior to mass spectrometry analysis, all powdered samples (100 mg) were extracted with 10 mL of acidified water (pH 2), and filtered through 0.22 μm nylon disk filters (Millipore, Billerica, MA, USA). 10 µL of the final solution were injected into the chromatograph. Analytical separation of peptides and proteins was performed on an Acquity BEH 300 C4 analytical column (100 mm × 2.1 mm internal diameter, 1.7 μm; Waters Corporation, Manchester, UK). A mobile phase consisting in a gradient program combining deionized water with 0.1% of acid formic as solvent A and acetonitrile with 0.1% of acid formic as solvent B was used. The initial conditions were 95% A and 5% B. A linear gradient was then established to reach 95% (*v*/*v*) of B at 15 min. Total run time was 25 min and post-delay time 5 min. Mobile phase flow rate was 0.4 mL/min.

After chromatographic separation, a high-resolution mass spectrometry analysis was carried out in positive electro spray ionization (ESI+ve). The gas used for desolvation (800 L/h) and Cone (25 L/h) was high-purity nitrogen. Spectra were recorded over the mass/charge (*m*/*z*) range 50–1200.

### 2.5. Animals and Experimental Design

Forty male obese homozygous (*fa*/*fa*) (O) and 40 lean heterozygous (*fa*/+) (L) Zucker rats (aged 5 weeks) with an initial mean body weight of 137 ± 2.5 and 120 ± 1.6 g, respectively, were allocated to eight different experimental groups (four obese and four lean groups, *n* = 10 rats each) within 2 different experiments RVR and GVR (raw vs. 4 day-germinated *V. radiata* dietary interventions). Two of the experimental groups in each experiment (an obese and a lean one) performed aerobic interval exercise (E) according to an established training protocol (OE, LE) while the two remaining were considered as sedentary (S) groups (OS, LS). The experiment lasted for 8 weeks, during which the animals were housed in a well ventilated, thermostatically controlled room (21 ± 2 °C). A reversed 12:12 light/dark cycle was implemented so the animals would perform the training protocol in darkness. Throughout the trial, animals had free access to type 2 water and consumed the experimental diet (Table 1) *ad libitum*. Food intake was recorded daily whereas body weight was measured once a week. At the end of experimental period, a glucose tolerance test was performed 48 h after the last training session by oral glucose overload. The animals were allowed to recover for 48 h prior being fasted for a further 8 h, anesthetized with xylazine/ketamine and sacrificed. Blood was collected by puncture of the abdominal aorta (with heparin as anticoagulant) and centrifuged at 1458× *g* for 15 min to separate plasma that was subsequently frozen in liquid nitrogen and stored at −80 °C. The liver was extracted, weighed, photographed for macroscopic studies, divided into various portions and immediately frozen in liquid nitrogen and stored at −80 °C. All experiments were undertaken according to Directional Guides Related to Animal Housing and Care [20] and all procedures were approved by the Animal Experimentation Ethics Committee of the University of Granada, Spain (Project Reference AGR4658).

### 2.6. Experimental Diet

The experimental diets were formulated following the guidelines of the American Institute of Nutrition (AIN-93M) [21], in order to meet the nutritional recommendations of adult rats [22], with whey (30%) and RVR or GVR (70%) as protein sources, to reach a 12% protein level (Table 1). A 0.5% methionine was added to fulfil the specific needs that the laboratory rats have respecting this specific amino acid. The percentage of dietary fibre added in the diet was established at 10% and was provided mainly from RVR or GVR flour while a small amount of cellulose was added in order to achieve the 10% level. The percentage of fat was set at 4%, using sunflower oil without any addition of saturated fat or other cholesterol source.

### 2.7. Exercise Protocol

The exercise groups followed a protocol of AIT five days a week during the eight weeks of the experimental period. The training protocol has been previously described by Kapravelou et al. [6], and was performed in a motorized treadmill specially designed for rats (Panlab Treadmills for five rats, LE 8710R). All sessions were performed during the dark cycle of the animals (active period). One week before the start of the study, the animals were adapted to the training procedures through a low intensity running protocol every day for 20 min in the treadmill at 18 m/min. The running sessions of 1 h started with a 10 min warm up at 40% VO_2_ max, and consisted of successive 4 min exercise periods at 65–80% of VO_2_ max, followed by 3 min recovery periods at 50–65% of VO_2_ max. The intensities and length of the training were gradually incremented every week (Table 2). To establish the velocity that would correspond to the VO_2_ max of each rat, a maximal incremental test was performed at the start of the study. A final incremental test was performed 96 h prior the end of the study to test the maximal aerobic capacity and physical performance achieved by the animals as a result of the intervention. The maximal incremental test was carried out following the protocol described by Wisløff et al. [23] and Clemente et al. [24] with slight modifications. Lactate concentrations were measured at the end of the incremental test in blood obtained from the animals’ tail (Lactate Pro, Arkray, The Netherlands).

### 2.8. Blood, Plasma and Liver Biochemical Analysis

Fasting blood glucose and glucose tolerance test were performed by oral glucose overload following the protocol described by Prieto et al. [25]. Blood glucose concentration from the animals’ tail was recorded at periods 0, 15, 30, 90 and 120 min after the glucose overload ingestion (BREEZE^®^ 2, Bayer Healthcare AG, Leverkusen, Germany), and the area under the curve (AUC) was determined. Biochemical parameters of lipid metabolism, and liver functionality were measured in plasma using a Shenzhen Mindray BS-200 Chemistry Analyzer (Shenzhen Mindray Bio-Medical Electronics, CO., LTD. Nanshan, Shenzhen, China) at the Bioanalysis Unit of the Scientific Instrumentation Centre (Biomedical Research Park, University of Granada, Granda, Spain). A portion of liver was lyophilized in order to determine the percentage of water. Hepatic lipids were extracted from the freeze-dried liver portion using the method described by Folch et al. [26] with slight modifications [7]. The extracted lipids were dissolved in 1 mL of 96% hexane to measure triglyceride and total-cholesterol content (Spinreact, S.A., Girona, Spain).

### 2.9. Macroscropic and Microscopic Liver Study

Liver area of the macroscopic image was estimated in all the liver images of the eight experimental groups assayed by morphometric study using the software Image Pro Plus 6.0 (Media Cybernetics, Inc., Rockville, MD, USA). A portion of liver was fixed in 10% phosphate-buffered formalin, dehydrated in ethanol, embedded in paraffin, and sectioned for histological examination using hematoxylin-eosin (HE), and Masson’s trichrome (MT) staining for general microscopy morphology and fibrosis development, respectively. Four different preparations of each staining were analyzed for each animal, and 10 animals were evaluated in each experimental group (*n* = 40 samples per group). Histological alterations were evaluated according to the following grading score: -, non-existent; +, mild; ++, mild/moderate; +++, moderate; ++++, abundant; +++++, severe.

### 2.10. Liver Protein Expression Analyses

Liver samples were homogenized in 20 mM Tris HCl buffer containing 0.1% Igepal, 100 mM Ethylene glycol-bis(2-aminoethylether)*-N,N,N′,N′*-tetraacetic acid, and a cocktail of protease inhibitors (Sigma, St. Louis, MO, USA). Liver homogenates were centrifuged at 13,000× *g* for 45 min, at 4 °C and supernatants were aliquoted and stored at −80 °C, until further use for western blot analysis. Protein concentration was measured by the method of Lowry et al. [27]. Equal amounts of total protein for each sample were loaded per well, subjected to 12% SDS-PAGE, and electrophoretically transferred to nitrocellulose membranes (Schleicher & Schuell, Dassel, Germany) by wet transfer at 100 V for 1 h using a Mini Trans-Blot cell system (Bio-Rad Laboratories, Hercules, CA, USA). Membranes were blocked using 5% non-fat dry powered milk dissolved in Tris-Buffered saline Tween-20 (TBST) for 90 min at room temperature (RT). The primary antibodies for 5′-AMP-activated protein kinase (AMPK) and phosphorylated-AMPK (pAMPK) (Cell Signaling Technology, Inc., Danvers, MA, USA) were used according to the manufacturer recommended dilutions (1:1000) and were incubated overnight at 4 °C. The membranes were then washed three times for 10 min with TBST, before incubation for 2 h at RT with secondary peroxidase conjugated goat anti-rabbit antibody (Sigma, St. Louis, MO, USA) diluted at 1:2000 in 5% skim-milk powder–TBST. Membranes were washed as before, and the bound antibodies were visualized by an ECL Pro system (PerkinElmer, Boston, MA, USA) using a Fujifilm Luminescent Analyzer LAS-4000 mini System (Fujifilm, Tokyo, Japan). pAMPK expression was determined in relation to AMPK expression. Results were expressed in relative density units.

### 2.11. Statistical Analyses

Time-repeated measurement analysis was applied to weekly food intake and body weight gain data as well as to blood glucose content after an oral glucose overload in order to analyze within subject effects (time) or within group effects (phenotype or AIT protocol) on the above parameters. The effect of phenotype (lean vs. obese corresponding to a *fa*/*+* or *fa*/*fa* genotype), AIT protocol, and dietary intervention with RVR or GVR on final body weight, aerobic capacity and physical performance, plasma and liver biochemical parameters, hepatic antioxidant enzyme activity, and protein expression was analyzed by 2 × 2 × 2 factorial ANOVA with phenotype (lean vs. obese), AIT protocol (sedentary vs. exercise) and dietary intervention (RVR vs. GVR) as main treatments. The use of a 2 × 2 × 2 factorial ANOVA is based on the potential interactions among the three interventions assayed (phenotype, exercise, and diet) being significant in our statistical model in addition to single effects of exercise or dietary treatment in each of the two phenotypes tested. To reinforce the potential integrative strength of the statistical model implemented, the *R*^2^ statistic has been included in the tables as a measure of goodness of fit of the model, given that the coefficient of determination indicates the proportion of variability in a data set that can be accounted by the statistical model. Results are given as mean values and pooled standard error of the mean. Bonferroni’s test was used to detect differences between treatment means. The analyses were performed with SAS, version 9.0 (SAS Institute Inc., Cary, NC, USA), and the level of significance was set at *p* < 0.05.

## 3. Results

### 3.1. Chemical Analysis

The composition in total, insoluble, soluble protein and non-protein N, fat, and ash content of RVR and GVR flours, together with the formulation and proximate composition of the different experimental diets is presented in Table 1. With regard to RVR flour, 81.6% of the total N content corresponded to soluble protein N, whereas 5.5% corresponded to soluble non-protein N, and 12.9% corresponded to insoluble N. Germination process caused a 7-fold increase in the content of soluble non-protein N, and a 1.4 and 1.6-fold decrease in the levels of insoluble and soluble protein N respectively. Germination caused a 1.2-fold increase in ash content without affecting total fat composition.

**Figure 1:**
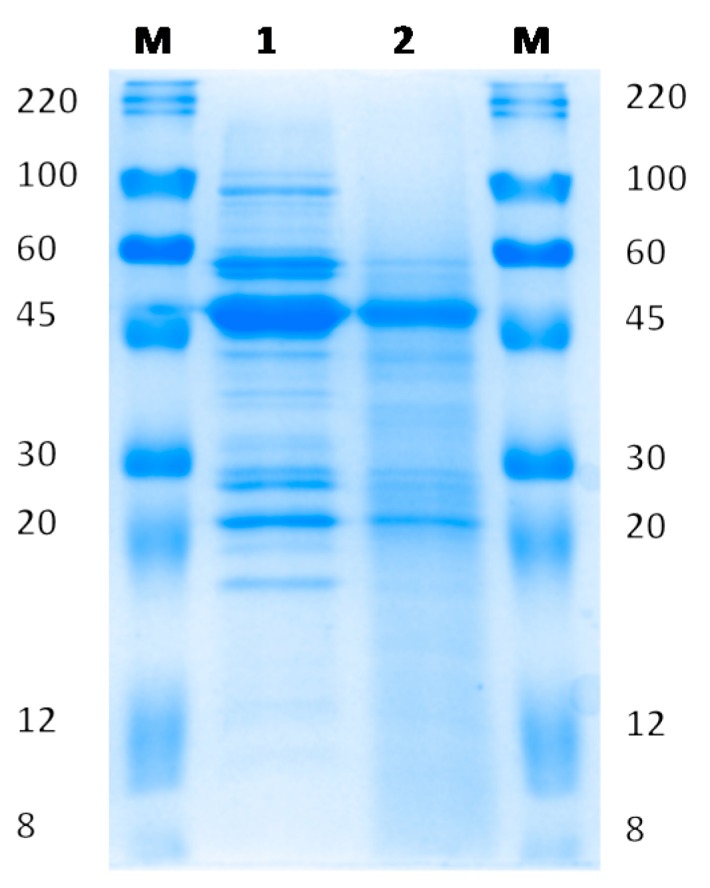
Detection processes of *V. radiata* protein digestion by germination process.Band pattern in SDS-PAGE. **M**, molecular weight markers; **1**, raw *V. radiata* protein extract; **2**, 4 day-germinated protein extract. The amount of Kjeldahl-N loaded per lane was 2.0 µg. The figure is representative of 3 independent analyses.

Figure 1 shows the changes in the SDS-PAGE pattern of proteins extracted from RVR and GVR flours. Germination process caused a reduction in density or the disappearance of main protein bands and the subsequent appearance of a smear of low molecular weight protein bands.

**Figure 2:**
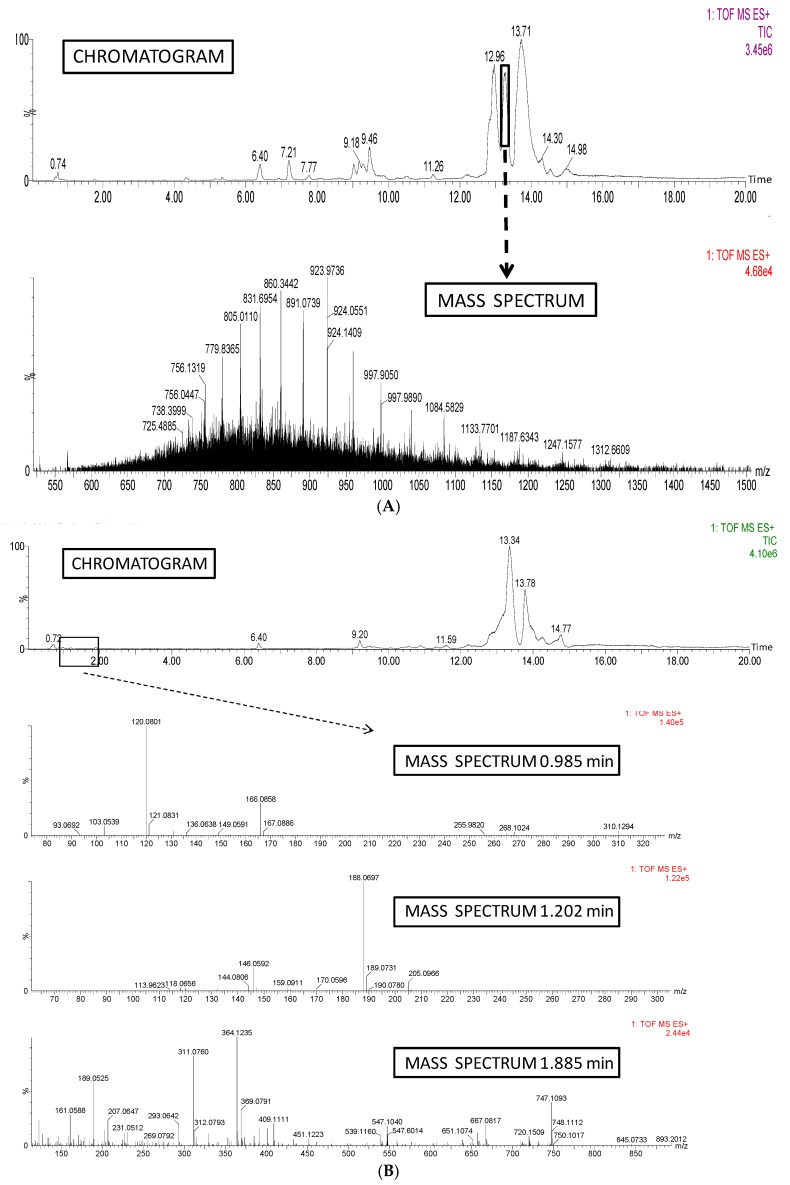
(**A**) Chromatogram of raw *V. radiata* protein and Mass Spectrum of major peak (13.270 min) of chromatogram; (**B**) Chromatogram of 4 day-germinated *V. radiata* protein after hydrolysis and Mass Spectrum of hydrolysis-derived peaks (0.985, 1.202, and 1.885 min, respectively) of chromatogram.

Figure 2A shows a chromatogram and a mass spectrum (Retention time 13.27 min) of RVR protein. Consistent with the complexity of the sample, a wide range of chromatographic peaks were detected. The presence of proteins was proved by high-resolution mass spectrometry as it is indicated in the mass spectrum example at 13.27 min. A typical protein profile for mass spectrometry was clearly obtained, due to the detection of multiple-charged ions provided by electro spray ionization of proteins present in the sample. In comparison to the former results, a chromatogram and a mass spectrum data of GVR protein are presented in Figure 2B. A simpler chromatogram was achieved, showing a 36% reduction in the area under the chromatogram that matched the disappearance or consistent decrease in the area of major peaks, and the minor appearance of new chromatographic peaks at 0.985, 1.202, and 1.883 min, respectively. The typical protein profile for mass spectrometry was obtained in the major peak (13.03 min), whereas the appearance of mono-charged ions that involved the presence of smaller molecules such as amino acids or peptides characterized the smaller peaks (0.985, 1.202, and 1.883 min) that ranged mass/charge (*m*/*z*) of 93–893.

### 3.2. Biological Analysis

#### 3.2.1. Food Intake and Body Weight Gain

**Figure 3:**
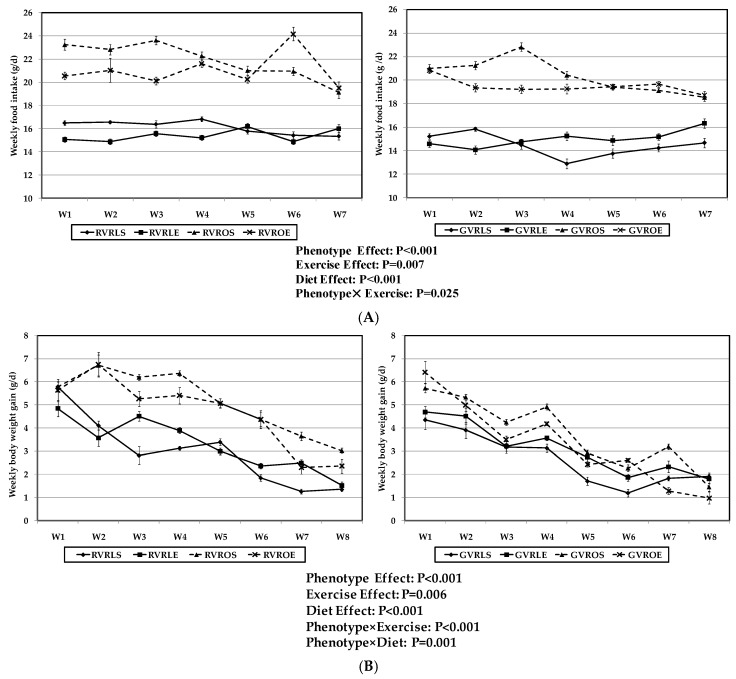
Effect of dietary treatment with raw or 4 day-germinated *V. radiata* and aerobic interval training protocol on food intake (**A**) and body weight gain (**B**) of lean and obese Zucker rats. (**A**) Weekly food intake (g DM/day); (**B**) Weekly body weight gain (g/day). DM, dry matter, RVR, raw *V. radiata*, GVR, 4 day-germinated *V. radiata*. Groups: LS, Lean (*fa*/+) sedentary rats, LE, Lean (*fa*/+) rats performing aerobic interval exercise, OS, Obese (*fa*/*fa*) sedentary rats, OE, Obese (*fa*/*fa*) rats performing aerobic interval exercise. W, week. Values are means ± SEM depicted by vertical bars (*n* = 10).

The effects of phenotype, AIT protocol and dietary treatment with RVR or GVR on weekly food intake and weekly body weight gain are presented in Figure 3. Both parameters were affected by phenotype with higher values found in the obese groups when compared with the lean ones. A significant effect of the AIT protocol was also observed, leading to decreased values of food intake (Figure 3A) and body weight gain (Figure 3B) in the groups that underwent the exercise training protocol when compared to their sedentary counterparts. Such reducing effects of exercise on food intake and weight gain were more pronounced in obese than in lean phenotype, thus resulting in a statistical interaction between the exercise intervention and the phenotype of rats (*p* = 0.025 and *p* < 0.0001, respectively).

With regard to the dietary treatment, GVR led to lower food intake and body weight gain when compared to RVR diet, although the effects on weight gain were more pronounced in the obese when compared to the lean animals, thus giving rise to a significant phenotype × diet interaction (*p* = 0.001).

#### 3.2.2. Aerobic Capacity and Physical Performance

The effects of phenotype, AIT protocol, and dietary treatment on aerobic capacity and physical performance of Zucker rats undergoing a maximal oxygen consumption incremental test, together with the effects on hematic parameters related to aerobic capacity are shown in Table 3. With the exception of VO_2_ max, all parameters related to aerobic capacity and physical performance derived from a maximal oxygen consumption incremental test were significantly affected by phenotype and exercise. Blood lactate content was 2-fold higher in sedentary obese rats compared to the lean groups, while it was only 1.3–1.5-fold higher in obese trained rats fed GVR and RVR, respectively (*p* < 0.001 for both phenotype and exercise effect). Significant effects of phenotype and exercise were also observed in running time, maximal speed and distance run that were lower in obese compared to lean animals and improved substantially in both groups as a result of the AIT protocol (all *p* < 0.001) Furthermore, a significant effect of exercise was observed on VO_2_ max that increased in both phenotypes (*p* < 0.001), although the effects were more pronounced in lean compared to obese animals, thus resulting in significant phenotype × exercise interaction (*p* < 0.001). Regarding the inclusion of RVR or GVR in the experimental diets, running time, maximal speed and distance run were enhanced by the GVR compared to RVR diet, with more pronounced effects in lean animals. All these, gave rise to significant diet effect (*p* < 0.001) and diet × phenotype interactions (*p* = 0.001 and *p* < 0.001 for running time and distance run, respectively). In addition, the parameters related to physical performance were differentially affected by exercise depending on the type of diet consumed. In this regard, the enhancing effect of exercise in both lean and obese animals was greatest in rats fed GVR, thus resulting in significant diet × exercise interaction (all *p* < 0.001). The AIT protocol assayed tended to increase all the hematic parameters related to aerobic capacity (Table 3). Such enhancing effect varied between lean and obese rats in most of the parameters studied, and gave rise to significant effect of exercise and phenotype × exercise interaction.

#### 3.2.3. Plasma Parameters of Glucose and Lipid Metabolism

**Figure 4:**
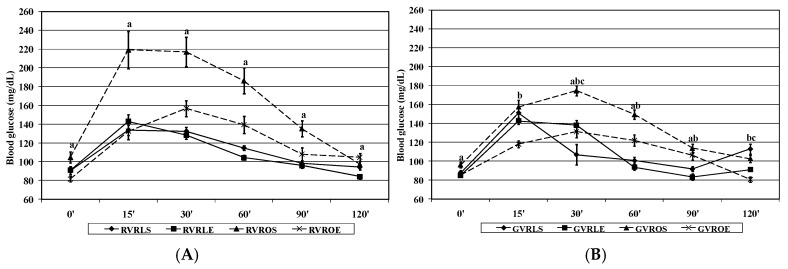
Effect of dietary treatment with raw or 4 day-germinated *V. radiata* and aerobic interval training protocol on blood glucose levels of lean and obese Zucker rats prior to or at different time points after oral glucose overload. (**A**) raw *V. radiata* (RVR), (**B**) 4 day-germinated *V. radiata* (GVR). Groups: LS, Lean (*fa*/+) sedentary rats, LE, Lean (*fa*/+) rats performing aerobic interval exercise, OS, Obese (*fa*/*fa*) sedentary rats, OE, Obese (*fa*/*fa*) rats performing aerobic interval exercise. Values are means ± SEM depicted by vertical bars (*n* = 10). The following notation is used to express significant differences (*p* < 0.05) between groups pointed out by Dunnet’s *t*-test: a, OS vs. LS, b, OE vs. LS, c, LE vs. LS.

The effects of phenotype, AIT protocol, and dietary treatment on blood and plasma biochemical parameters related to glucose and lipid metabolism are shown in Figure 4 and Table 4. A significant effect of phenotype was observed on all the parameters analyzed that were higher in obese when compared to lean rats. The AIT protocol assayed caused a significant reduction in blood glucose and AUC, as well as in plasma lipid parameters, with a more pronounced effect in obese animals, giving rise to a significant phenotype × exercise interaction. AUC values were also lower in animals fed the GVR when compared to RVR diet, and such dietary effect was modulated by phenotype, thus resulting in significant phenotype × diet interaction (*p* < 0.001). Likewise, diet significantly affected the influence of exercise on AUC, with a less evident effect in GVR compared to RVR, resulting in a significant diet × exercise interaction. All these effects were reflected in different trends of evolution in blood glucose values after an oral glucose overload in GVR compared to RVR (Figure 4), with lower levels of peak blood glucose and time to return to basal levels by the obese animals in the former dietary treatment.

Total-, LDL-, and HDL-cholesterol were also significantly affected by diet and exercise, with significantly lower values found in GVR vs. RVR-fed, and in trained vs. untrained animals. However, these effects were only apparent in obese but not in lean animals, thus giving rise to significant phenotype × diet and phenotype × exercise interactions.

#### 3.2.4. Hepatic Functionality and Antioxidant Activity

The effects of phenotype, AIT protocol, and dietary treatment on different parameters of liver weight, chemical composition and functionality are presented in Table 5. There was a significant effect of phenotype on liver weight, surface, total fat, and triglyceride content that were all higher in the obese when compared to the lean groups. A significant effect of the AIT protocol was observed for most of the former parameters, leading to an exercise-derived reduction only among the obese groups, thus giving rise to significant phenotype × exercise interaction. A diet effect was observed for total weight and surface area of the liver that were higher in RVR than in GVR. The GVR caused a clear reduction in fat and triglyceride content of obese animals compared to the RVR counterparts, but no effect was observed in lean animals thus resulting in significant phenotype × diet interaction. In relation to hepatic cholesterol content, a significant diet effect was observed related to lower levels of this component in GVR- compared to RVR-fed animals, without any clear effect of phenotype in this parameter. The training protocol was more efficient at reducing hepatic cholesterol in rats fed GVR compared to those fed RVR, and in lean vs. obese animals, thus giving rise to significant exercise × diet and exercise × phenotype interactions.

Plasma Aspartate Transferase (AST), Alanine Transferase (ALT), and Alkaline Phosphatase (ALP) activities were used as markers of liver function. A significant phenotype effect was observed on these parameters leading to higher values in the obese groups when compared to the lean ones. Exercise managed to decrease the activities of AST, ALT, and ALP among the obese groups, but a significant increase in ALP was observed for the lean animals. There was also a significant effect of diet in AST and ALT activities with lower values found in GVR than in RVR animals. The effects of diet were more obvious in obese than in lean animals, thus giving rise to a significant phenotype × diet interaction. With regard to the hepatic antioxidant enzyme activities, the influence of the different interventions assayed on the outcomes studied varied widely and did not reach significant biological importance.

#### 3.2.5. Liver Histology

**Figure 5:**
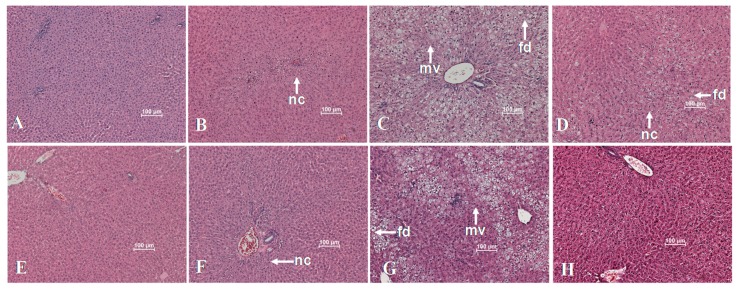
Effect of dietary treatment with raw or 4 day-germinated *V. radiata* and AIT protocol on liver histology (hematoxylin-eosin stain) of lean and obese Zucker rats. (**A**) RVR-LS, (**B**) RVR-LE, (**C**) RVR-OS, (**D**) RVR-OE, (**E**) GVR-LS, (**F**) GVR-LE, (**G**) GVR-OS, (**H**) GVR-OE. RVR, raw *V. radiata*, GVR, 4 day-germinated *V. radiata*. Groups: LS, Lean (*fa*/+) sedentary rats, LE, Lean (*fa*/+) rats performing aerobic interval exercise, OS, Obese (*fa*/*fa*) sedentary rats, OE, Obese (*fa*/*fa*) rats performing aerobic interval exercise. Photographs are representative of livers of 10 different rats for each experimental group. nc, necrosis, mv, microvesicular steatosis, fd, fatty droplet accumulation.

There was a clear phenotype effect on liver histopathological parameters related to the development of NAFLD (Figure 5, Table 6) among the different experimental groups. This resulted in a manifestation of micro and macrovesicular steatosis signs as well as lipogranulomas and portal inflammation on the obese but not in lean animals. Exercise could partly reverse these alterations, leading to a decrease of micro and macrovecicular steatosis, fatty droplets, lipogranulomas and portal inflammation, although such effects were more prominent in GVR than in RVR-fed animals. Therefore, the combination of the GVR diet and the AIT protocol led to improved NAFLD liver histology when compared to the combination of AIT protocol and RVR. However, despite the above mentioned improvements, exercise caused the appearance of necrosis in GVR but not in RVR-fed animals although no signs of fibrosis or multinucleic cells were detected in any of the experimental groups tested.

#### 3.2.6. Protein Expression

**Figure 6:**
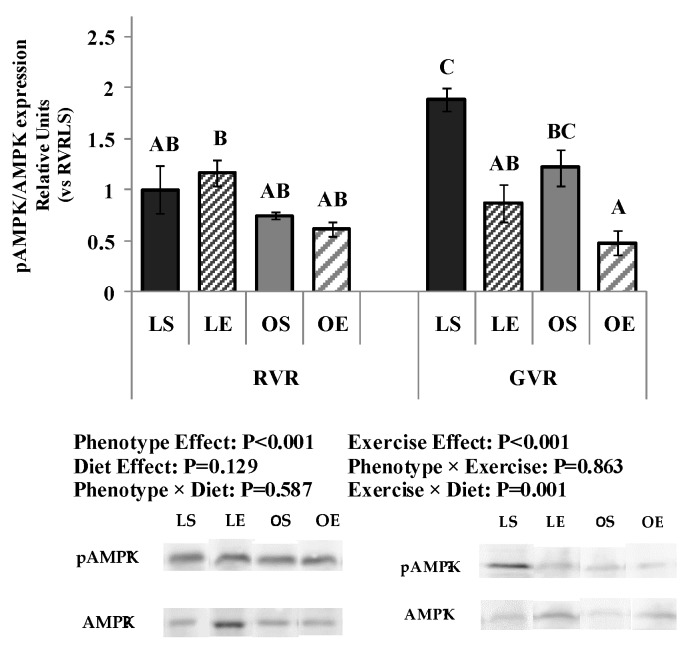
Effect of dietary treatment with raw or 4 day-germinated *V. radiata* and aerobic interval training protocol on AMPKα/pAMPKα protein expression in the liver of lean and obese Zucker rats measured by Western blot analysis. AMPK, 5′-AMP-activated protein kinase; phosphorylated-AMPK, pAMPK. RVR, raw *V. radiata*, GVR, 4 day-germinated *V. radiata*. Groups: LS, Lean (*fa*/+) sedentary rats, LE, Lean (*fa*/+) rats performing aerobic interval exercise, OS, Obese (*fa*/*fa*) sedentary rats, OE, Obese (*fa*/*fa*) rats performing aerobic interval exercise. Immunoblots are representative of liver homogenates of eight different rats for each experimental group; all samples were derived at the same time and processed in parallel. The amount of sample loaded per lane was 100 µg of protein. Levels of pAMPK were normalized to the total AMPK. Densitometric analysis values represented in the graphs are means ± SEM depicted by vertical bars (*n* = 8). Means without a common letter differ, *p* < 0.05.

The effects of phenotype, AIT protocol, and dietary treatment on protein expression of AMPK and pAMPK are shown in Figure 6. pAMPK/AMPK ratio was significantly affected by phenotype, showing lower values in obese compared to lean rats (*p* < 0.001). GVR diet tended to increase AMPK phosphorylation compared to RVR, although the results did not reach statistical significance (*p* = 0.129). With regard to the AIT protocol, it decreased the degree of AMPK phosphorylation in all the experimental groups with the exception of lean animals fed RVR, thus resulting in significant exercise effect (*p* < 0.001) and exercise × diet interaction (*p* = 0.001).

## 4. Discussion

In the present study, we aimed to study the beneficial effects of a mixed intervention with aerobic interval training protocol and RVR or GVR diets on plasma and liver parameters of hepatic functionality using the obese Zucker rat experimental animal model. Obese rats exhibited higher food intake and body weight, and suffered significant alterations in plasma lipid profile, glucose tolerance test, liver tests and histology. Exercise increased the aerobic capacity of both rat phenotypes and diminished the severity of MetS alterations, especially those related to glucose and lipid metabolism. Consumption of germinated *V. radiata* in combination with the AIT protocol was more efficient than the raw legume at reversing the alterations in aerobic capacity, glucose and lipid metabolism, and liver histology and functionality.

The germination process selected to obtain a natural protein hydrolyzate from *V. radiata* was effective at breaking down proteins in small molecular weight peptides such as shown by both chemical and electrophoretic methods. The results were further confirmed by mass spectrometry that showed the appearance of newly formed low molecular weight peptides with low retention times. Our results are in agreement with those reported by Urbano et al. [9] in pea, in which 3 or 4 day-germination induced the activation of endogenous proteases and the consequent hydrolysis of legume storage proteins giving rise to a significant increase in the content of soluble non-protein N and considerable changes in the electrophoretic pattern of pea proteins. Legume protein hydrolyzates have been shown to be effective as functional foods for the treatment of alterations included in the MetS [7] due to both their content in biologically active peptides and other components like dietary fiber, phytic acid or polyphenols.

Obese Zucker rats are known to present a genetic defect in leptin receptor that causes the development of hyperphagia as well as other metabolic disturbances [18]. A decrease in food intake of rats similar to what has been found in the present study has been reported resulting from low-moderate intensity exercise associated to the production of corticotrophin-releasing hormone [28]. Furthermore, food intake can be affected by legume consumption, especially when prolonged periods of germination (4–6 days) are used, due to the appearance of compounds responsible for the loss of organoleptic properties [10].

Physical performance was always lower in obese when compared to lean Zucker rats due to the severe metabolic disturbances, impaired skeletal muscle perfusion, and muscular atrophy inherent to this experimental model. Moreover, low intrinsic aerobic capacity in rats has been related to lower energy expenditure and reduced whole body and hepatic mitochondrial lipid oxidation, which can induce a higher susceptibility to dietary-induced hepatic steatosis [29]. As a result of the AIT protocol, there was a clear improvement in the aerobic capacity and physical performance parameters of lean and obese rats that was more pronounced when combined with the consumption of GVR diet. Furthermore, under our experimental conditions, the higher physical performance and aerobic capacity of trained Zucker rats was related to significant changes in glucose and lipid metabolism as well as to improved hepatic histology and function altered in NAFLD. It is worth mentioning that the complex chemical changes in protein or polyphenolic profile brought about by germination of *V. radiata* [30] resulted in generally improved health status of the animals when compared to raw controls, thus reassuring the potential of the former functional ingredient in the dietary treatment of several alterations related to the MetS.

The development of MetS in obese Zucker rats was associated to alterations in glucose and lipid metabolism as reported by other authors [31,32]. Our results showed a positive action of AIT protocol in plasma lipid profile, and point out to improved insulin sensitivity of trained obese animals exemplified by plasma AUC after an oral glucose overload. The beneficial action exerted by the AIT protocol on the above-mentioned parameters of glucose and lipid metabolism was more obvious in obese rats fed GVR vs. RVR, and especially on plasma AUC. The antidiabetic properties of *V. radiata* L. are mainly assigned to different non-nutritional components like d-*chiro*-inositol and the synergistic effects of its two major phenolics: vitexin and isovitexin [33], as well as to the slow-digestible nature of its complex carbohydrates which is reflected in their low glycemic index [34]. The stronger action of GVR vs. RVR could be related to changes in non-nutritional components that are present in higher concentrations in GVR than in RVR [33]. The hypolipidemic effects of *V. radiata* are thought to arise from its phytosterol and dietary fiber content that act in the prevention of cholesterol biosynthesis and absorption and cecal fermentation [12]. Nevertheless, under our experimental conditions, the administration of GVR experimental diets could not reverse by itself the elevated levels of plasma cholesterol and triglycerides, although the dietary inclusion of GVR legume flour in association with exercise did induce a greater decrease in the former parameters compared to RVR, thus pointing to a synergistic effect of both interventions.

In the present study, using the Non Alcoholic Steatohepatitis grading score described by Brunt et al. [35], a value of 2 (moderate) in the necroinflammatory grading system can be ascribed to the livers of obese sedentary rats based on the appearance of several histological lesions, like steatosis seen in up to or greater than 66% of the hepatocytes, cell ballooning, presence of lipogranulomas and moderate portal inflammation. Such observation related to phenotype was correlated to the significantly higher hepatic total fat and TG but not cholesterol content. Obese Zucker rats have been described to present increased levels of hepatic total lipids and triglyceride content. However, there appears to be no general agreement about hepatic cholesterol content, since some authors have not found significant differences between phenotypes [36] as in our results, whereas others found greater values in the obese diabetic Zucker rats compared to their lean counterparts [37]. The AIT protocol caused a significant improvement in several of the histopathological lesions, thus decreasing the score value to 1 (mild) in the necroinflammatory grading system. Our present results are in agreement with the improvement of several independent predictors of NASH described by Ballestri et al. [38] such as insulin resistance, plasma total cholesterol and ALT as well as to a decrease in plasma triglycerides and hepatic total fat and triglyceride content. In this way, training protocols using treadmill or wheel ergometers are good interventions to decrease the hepatic levels of total lipids and triglycerides in rats [6,39]. However, we found very mild effects of the training protocol assayed on hepatic cholesterol, and only a consistent exercise-induced decrease in the above mentioned parameter was observed in lean or obese animals fed GVR. It can be hypothesized that changes in plasma cholesterol levels of Zucker rats following exercise are mediated by extra-hepatic mechanisms [40] or else, that pathways involved in the metabolism of cholesterol and triglycerides are differentially affected by the interventions tested, giving rise to the interactions found under our experimental conditions.

In addition, the administration of GVR diet led to a greater improvement in hepatic status compared to RVR as demonstrated by the lower liver weight and area of the obese animals that consumed this specific diet. Such increased potential of the GVR diet may be due to the compositional changes that take place during germination such as the increase in polyphenol, antioxidant capacity and vitamin C levels of *V. radiata* sprouts [30,41]. Cholesterol content in the liver of GVR-fed animals was lower compared to the RVR-fed ones, thus confirming the hypothesis that germination is an effective biotechnological process with strong beneficial effects on the composition of healthy bioactive compounds present in legumes. The administration of the GVR experimental diet in combination with the AIT protocol also led greater improvements in hepatic histology and plasma parameters of hepatic functionality of obese trained groups. Such results emphasize the advantages, not only of exercise, as strong lifestyle intervention strategy for the prevention and treatment of NAFLD, but also of legume-based foodstuffs and certain biotechnological treatments like germination to potentiate the benefits of other lifestyle strategies actually in use for the treatment of the above mentioned disease.

Activation of AMPK depends on the ADP:ATP ratio, and it is reduced in the liver of obese Zucker rat due to an excess of energetic substrates entering this tissue [18]. In addition, AMPK activity can be inhibited by insulin and glucose in several tissues, as it would be the case with the hyperinsulinemia characteristic of this experimental animal model. The AIT protocol assayed tended to inhibit the beneficial AMPK activation induced by consumption of GVR. A finding that points out to potential differences in the way that the lifestyle interventions assayed affected the entrance of energetic substrates to the liver and their metabolization by this organ as well as to the strong influence of the genetic metabolic disorder suffered by obese Zucker rats in which the presence of numerous factors capable of interacting with our two interventions have modified the response of liver in terms of signal protein activation.

## 5. Conclusions

The beneficial effects on insulin resistance, hyperlipidemia, and liver tests and histology exerted by the combination of the two lifestyle strategies assayed (protein hydrolyzates from germinated *V. radiata* and physical exercise) represented an efficient and feasible complement for the treatment of MetS.

Since patients affected with MetS have increased risk of developing NAFLD, we put forward the hypothesis that lifestyle interventions based on dietary treatment with functional ingredients in combination with a specific training protocol are an alternative or complementary treatment to improve the metabolic alterations caused by the disease. Therefore, liver histological and functionality analyses may contribute to a better knowledge about the pathophysiology of NAFLD and to establish an efficient treatment based on nutritional and healthy lifestyle strategies to improve metabolic syndrome and derived hepatic dysfunction.

## Figures and Tables

**Table 1:** Composition of raw and 4 day-germinated *V. radiata* flours and experimental diet formulation.

***V. radiata* Flour**	**RVR**	**GVR**
Moisture (g/kg)	83.0	88.4
Total N (g/kg DM)	34.4	42.0
Insoluble N (%)	12.9	9.4
Soluble Protein N (%)	81.6	50.9
Soluble Non Protein N (%)	5.5	39.7
Total fat (g/Kg)	17.0	14.6
Ash (g/kg DM)	37.1	43.4
**Diet Formulation (g/kg)**	**RVR**	**GVR**
Raw *V. radiata*	367	-
Germinated *V. radiata*	-	333
Whey Protein	56	56
Sucrose	100	100
Cellulose	11	21
Methionine	5	5
Sunflower Oil	40	40
Mineral premix	35	35
Vitamin premix	10	10
Choline bitartrate	2.5	2.5
Starch	427	411

RVR, Raw *V. radiata*; GVR, 4 day-germinated *V. radiata*; DM, dry matter.

**Table 2:** Details of the aerobic interval training (AIT) protocol.

Week (5 Days/Week)	Work Time (min/Day)	% VO_2_ max
1	45′	50%→3 min
		65%→4 min
2	50′	55%→3 min
		70%→4 min
3	50′	60%→3 min
		75%→4 min
4	55′	60%→3 min
		75%→4 min
5–8	60′	65%→3 min
		80%→4 min

VO_2_ max, maximal oxygen consumption.

**Table 3:** Effect of diet, phenotype, and aerobic interval training protocol on aerobic capacity, physical performance and related hematic parameters of Zucker rats.

	RVR				GVR				*R*^2^	Phenotype Effect	Diet Effect	Exercise Effect	Phenotype× Diet	Phenotype × Exercise	Exercise × Diet
	Lean		Obese		Lean		Obese								
	SED	EXC	SED	EXC	SED	EXC	SED	EXC							
Lactate (mmol/L)	6.65^A^ (0.44)	5.07^A^ (0.68)	13.62^C^ (0.68)	10.21^B^ (0.57)	7.53^A^ (0.38)	6.91^A^ (0.56)	13.94^C^ (0.74)	10.08^B^ (0.69)	0.769	*p* < 0.001	*p* = 0.077	*p* < 0.001	*p* = 0.121	*p* = 0.002	*p* = 0.744
VO_2_max (mL/min/kg^0.75^)	18.5^A^ (1.25)	25.4^E^ (0.85)	21.4^BCD^ (0.15)	22.2^CD^ (0.28)	19.1^AB^ (0.69)	25.9^E^ (0.38)	20.6^ABC^ (0.38)	23.4^CD^ (0.70)	0.668	*p* = 0.440	*p* = 0.469	*p* < 0.001	*p* = 0.654	*p* < 0.001	*p* = 0.277
Running Time (min)	12.5^A^ (0.82)	21.3^D^ (0.77)	9.51^A^ (0.52)	13.9^BC^ (1.05)	14.2^BC^ (0.36)	27.1^E^ (0.91)	8.75^A^ (0.35)	16.3^C^ (0.51)	0.915	*p* < 0.001	*p* < 0.001	*p* < 0.001	*p* = 0.001	*p* < 0.001	*p* < 0.001
Maximal Speed (cm/s)	53.8^B^ (2.43)	81.6^D^ (2.49)	41.5^A^(1.56)	51.4^B^ (3.24)	57.8^BC^ (1.02)	96.0^E^ (2.81)	41.2^A^ (0.92)	63.4^C^ (1.50)	0.908	*p* < 0.001	*p* < 0.001	*p* < 0.001	*p* = 0.195	*p* < 0.001	*p* < 0.001
Distance (cm)	28,434^B^ (2459)	65,985^D^ (3928)	17,260^A^ (1352)	29,931^BC^ (3437)	32,675^BC^ (2749)	94,789^E^ (4190)	16,593^A^ (635)	39,616^C^ (1917)	0.931	*p* < 0.001	*p* < 0.001	*p* < 0.001	*p* < 0.001	*p* < 0.001	*p* < 0.001
RBC (×10^6^/μL)	8.77^A^ (0.09)	9.57^AB^ (0.34)	9.29^AB^ (0.31)	9.42^AB^ (0.44)	9.16^AB^ (0.09)	9.98^B^ (0.19)	8.92^AB^ (0.36)	8.57^A^ (0.28)	0.243	*p* = 0.086	*p* = 0.583	*p* = 0.075	*p* = 0.006	*p* = 0.009	*p* = 0.443
HGB (g/dL)	13.2^AB^ (0.12)	16.0^CD^ (0.58)	13.8^ABC^ (0.50)	16.1 ^D^ (0.69)	13.8^ABC^ (0.15)	15.3^CD^ (0.35)	11.8^A^ (0.84)	14.5^BCD^ (0.31)	0.475	*p* = 0.125	*p* = 0.015	*p* < 0.001	*p* = 0.003	*p* = 0.462	*p* = 0.509
HCT (%)	43.3^A^ (0.43)	49.6^BC^ (1.75)	46.2^AB^ (1.58)	50.5^BC^ (2.32)	45.5^AB^ (0.57)	51.4^C^ (1.10)	42.8^A^ (1.91)	42.4^A^ (1.35)	0.489	*p* = 0.012	*p* = 0.290	*p* < 0.001	*p* < 0.001	*p* = 0.012	*p* = 0.131
MCV (fL)	49.6^A^ (0.15)	51.8^B^ (0.14)	49.7^A^ (0.28)	52.0^B^ (0.31)	49.7^A^ (0.23)	51.5^B^ (0.23)	49.8^A^ (0.26)	51.1^B^ (0.23)	0.670	*p* = 0.693	*p* = 0.101	*p* < 0.001	*p* = 0.367	*p* = 0.427	*p* = 0.017
MCH (pg)	15.1^A^ (0.07)	17.1^C^ (0.29)	14.8^A^ (0.24)	16.1^B^ (0.24)	15.1^A^ (0.08)	15.3^AB^ (0.14)	14.8^A^ (1.09)	17.0^C^ (0.30)	0.681	*p* = 0.866	*p* = 0.165	*p* < 0.001	*p* < 0.001	*p* = 0.019	*p* = 0.117
RDW (fL)	28.5^A^ (0.09)	30.4^B^ (0.17)	30.9^B^ (0.30)	34.6^C^ (1.01)	29.8^AB^ (0.21)	29.8^AB^ (0.30)	29.8^AB^ (0.21)	32.9^C^ (0.68)	0.714	*p* < 0.001	*p* = 0.087	*p* < 0.001	*p* = 0.002	*p* < 0.001	*p* = 0.024

^A,B,C,D,E^ Results are mean of 10 rats. Means within the same line with different superscripts differ significantly (*p* < 0.05), data in parenthesis represent standard error of the mean. RVR, Raw *V. radiata*, GVR, Germinated *V. radiata*, SED, sedentary rats, EXC, rats performing a protocol of aerobic interval exercise, *R*^2^, coefficient of determination, VO_2_ max, Maximum Oxygen Volume. RBC, Red blood cells, HGB, Hemoglobin, HCT, Hematocrit, MCV, Mean corpuscular volume, MCH, Mean corpuscular hemoglobin, RDW, Red cell distribution width.

**Table 4:** Effect of diet, phenotype, and aerobic interval training protocol on blood and plasma parameters related to glucose and lipid metabolism.

	RVR				GVR				*R*^2^	Phenotype Effect	Diet Effect	Exercise Effect	Phenotype × Diet	Phenotype × Exercise	Exercise × Diet
	Lean		Obese		Lean		Obese								
	SED	EXC	SED	EXC	SED	EXC	SED	EXC							
Glucose (mg/dL)	87.0^A^ (2.2)	91.2^A^ (2.3)	104.9^B^ (5.9)	81.8^A^ (3.4)	86.2^A^ (2.7)	85.0^A^ (1.7)	93.8^AB^ (2.0)	85.5^A^ (2.5)	0.365	*p* = 0.044	*p* = 0.088	*p* = 0.002	*p* = 0.926	*p* < 0.001	*p* = 0.285
AUC (arbitrary units)	3611^BC^ (129)	2433^AB^ (233)	7226^D^ (549)	5878^D^ (61)	1965^A^ (178)	2722^AB^ (255)	4312^C^ (333)	3445^BC^ (396)	0.770	*p* < 0.001	*p* < 0.001	*p* = 0.002	*p* < 0.001	*p* = 0.039	*p* = 0.007
T-Cholesterol (mg/dL)	72.5^A^ (2.2)	83.0^A^ (3.6)	223.9^D^ (10.1)	172.3^C^ (5.7)	79.4^A^ (1.5)	88.9^A^ (2.7)	207.3^D^ (11.5)	120.1^B^ (6.8)	0.936	*p* < 0.001	*p* < 0.001	*p* < 0.001	*p* < 0.001	*p* < 0.001	*p* = 0.011
LDL-Cholesterol (mg/dL)	3.2^A^ (0.3)	12.8^B^ (1.8)	39.5^D^ (4.2)	27.1^C^ (3.2)	3.4^A^ (0.3)	7.9^AB^ (0.8)	36.6^D^ (3.3)	16.0^B^ (1.6)	0.849	*p* < 0.001	*p* = 0.001	*p* = 0.001	*p* = 0.099	*p* < 0.001	*p* = 0.018
HDL-Cholesterol (mg/dL)	26.0^A^ (0.4)	29.1^AB^ (1.7)	59.9^D^ (2.2)	46.1^C^ (3.7)	27.6^AB^ (0.5)	28.1^AB^ (0.9)	44.3^C^ (3.6)	35.1^B^ (2.6)	0.803	*p* < 0.001	*p* < 0.001	*p* < 0.001	*p* < 0.001	*p* < 0.001	*p* = 0.701
Triglycerides (mg/dL)	31.2^A^ (5.0)	23.4^A^ (6.0)	468.9^C^ (58.7)	353.4^B^ (39.1)	28.8^A^ (2.3)	40.2^A^ (7.8)	470.1^C^ (71.4)	303.6^B^ (17.9)	0.883	*p* < 0.001	*p* = 0.603	*p* < 0.001	*p* = 0.341	*p* < 0.001	*p* = 0.629

^A,B,C,D^ Results are mean of 10 rats. Means within the same line with different superscripts differ significantly (*p* < 0.05), data in parenthesis represent standard error of the mean. RVR, Raw *V. radiata*, GVR, Germinated *V. radiata*, SED, sedentary rats, EXC, rats performing a protocol of aerobic interval exercise, *R*^2^, coefficient of determination, AUC, Area under the curve.

**Table 5:** Effect of phenotype, diet, and aerobic interval training protocol on liver weight, chemical composition, and functionality.

	RVR				GVR				*R*^2^	Phenotype Effect	Diet Effect	Exercise Effect	Phenotype × Diet	Phenotype × Exercise	Exercise × Diet
	Lean		Obese		Lean		Obese								
	SED	EXC	SED	EXC	SED	EXC	SED	EXC							
Weight (g FW)	8.6^A^ (0.18)	9.7^A^ (0.64)	17.4^D^ (0.75)	14.7^C^ (0.94)	9.2^A^ (0.34)	10.4^A^ (0.58)	14.1^BC^ (0.71)	12.4^B^ (0.31)	0.832	*p* < 0.001	*p* = 0.001	*p* = 0.219	*p* < 0.001	*p* = 0.001	*p* = 0.638
Surface (cm^2^)	14.2^A^ (0.51)	14.0^A^ (0.65)	24.0^D^ (0.61)	18.1^C^ (0.47)	14.9^AB^ (0.34)	15.4^AB^ (0.43)	18.2^C^ (0.46)	16.9^BC^ (0.91)	0.755	*p* < 0.001	*p* = 0.002	*p* < 0.001	*p* < 0.001	*p* < 0.001	*p* = 0.002
Moisture (%)	69.3^D^ (0.15)	69.3^D^ (0.13)	58.2^A^ (1.55)	64.5^BC^ (0.82)	69.4^D^ (0.09)	67.7^CD^ (0.50)	63.0^B^ (0.94)	67.1^CD^ (0.30)	0.671	*p* < 0.001	*p* = 0.026	*p* = 0.002	*p* = 0.001	*p* > 0.001	*p* = 0.149
Fat (g/100 g DM)	1.52^A^ (0.33)	3.05^A^ (0.37)	21.9^B^ (2.09)	6.09^A^ (2.12)	3.58^A^ (0.28)	6.93^A^ (0.22)	17.9^B^ (1.53)	6.15^A^ (0.44)	0.714	*p* < 0.001	*p* = 0.512	*p* < 0.001	*p* = 0.020	*p* < 0.001	*p* = 0.151
Triglycerides (mg/g DM)	0.59^A^ (0.08)	0.90^A^ (0.24)	17.99^C^ (0.87)	1.72^A^ (1.75)	0.25^A^ (0.06)	0.37^A^ (0.08)	12.4^B^ (1.41)	0.95^A^ (0.33)	0.802	*p* < 0.001	*p* = 0.209	*p* < 0.001	*p* = 0.008	*p* < 0.001	*p* = 0.020
Cholesterol (mg/g DM)	6.59^A^ (0.34)	6.40^A^ (0.18)	4.95^AB^ (0.32)	6.85^A^ (0.62)	5.46^A^ (0.10)	4.60^B^ (0.21)	4.77^B^ (0.21)	4.51^B^ (0.34)	0.470	*p* = 0.061	*p* < 0.001	*p* = 0.567	*p* = 0.683	*p* = 0.011	*p* = 0.008
**Liver Function Plasma Markers**															
AST (U/L)	95.4^C^ (5.34)	61.6^A^ (2.35)	178.0^D^ (7.58)	96.1^C^ (9.91)	67.3^AB^ (4.13)	57.9^A^ (2.94)	104.4^C^ (11.20)	88.8^BC^ (6.21)	0.826	*p* < 0.001	*p* < 0.001	*p* < 0.001	*p* = 0.002	*p* = 0.001	*p* < 0.001
ALT (U/L)	37.3^A^ (2.66)	28.6^A^ (1.94)	160.8^D^ (11.45)	59.0^B^ (6.38)	35.5^A^ (4.05)	26.6^A^ (2.42)	115.4^C^ (13.13)	25.6^A^ (1.83)	0.922	*p* < 0.001	*p* < 0.001	*p* < 0.001	*p* < 0.001	*p* < 0.001	*p* = 0.379
ALP (U/L)	99.0^AB^ (2.69)	152.6^CD^ (7.57)	268.2^F^ (11.33)	180.2^DE^ (8.30)	80.8^A^ (2.52)	140.7^C^ (3.43)	201.8^E^ (16.44)	124.6^BC^ (4.92)	0.892	*p* < 0.001	*p* < 0.001	*p* = 0.008	*p* < 0.001	*p* < 0.001	*p* = 0.364

^A,B,C,D,E,F^ Results are mean of 10 rats. Means within the same line with different superscripts differ significantly (*p* < 0.05), data in parenthesis represent standard error of the mean. RVR, Raw *V. radiata* diet, GVR, Germinated *V. radiata* diet, SED, sedentary rats, EXC, rats performing a protocol of aerobic interval exercise, *R*^2^, coefficient of determination, FW, fresh weight, DM, dry matter, AST, aspartate aminotransferase, ALT, alanine transaminase, ALP, Alkaline Phosphatase.

**Table 6:** Effect of phenotype, diet, and aerobic interval training protocol on liver histology of Zucker rats.

	RVR				GVR			
	Lean		Obese		Lean		Obese	
	SED	EXC	SED	EXC	SED	EXC	SED	EXC
Microvescicularsteatosis	-	-/+	++++/+++++	+++/++++	-	+/++	++++/+++++	+/++
Fatty droplets	-	-	++++	++/+++	-	-	++++	+
Multinucleic cells	-	-	-	-	-	-	-	-
Lipogranulomas	-	+	++/+++	++/+++	++	++	++	+/++
Portal Inflammation	-/+	+/++	+++	++	+/++	+/++	+++	++
Necrosis	-	+	-	+	-	+/++	-	++
Fibrosis	-	-	-	-	-	-	-	-

RVR, Raw *V. radiata*, GVR, Germinated *V. radiata*, SED, sedentary rats, EXC, rats performing a protocol of aerobic interval exercise. Grading score of the histological alterations: -, non-existent; +, mild; ++, mild/moderate; +++, moderate; ++++, abundant; +++++, severe.
